# Predictors of mother–child interaction quality and child attachment security in at-risk families

**DOI:** 10.3389/fpsyg.2014.00898

**Published:** 2014-08-20

**Authors:** Simona De Falco, Alessandra Emer, Laura Martini, Paola Rigo, Sonia Pruner, Paola Venuti

**Affiliations:** Università degli Studi di TrentoRovereto, Italy

**Keywords:** attachment, emotional availability, psychosocial risk, parenting, psychosocial prevention

## Abstract

Child healthy development is largely influenced by parent–child interaction and a secure parent–child attachment is predictively associated with positive outcomes in numerous domains of child development. However, the parent–child relationship can be affected by several psychosocial and socio-demographic risk factors that undermine its quality and in turn play a negative role in short and long term child psychological health. Prevention and intervention programs that support parenting skills in at-risk families can efficiently reduce the impact of risk factors on mother and child psychological health. This study examines predictors of mother–child interaction quality and child attachment security in a sample of first-time mothers with psychosocial and/or socio-demographic risk factors. Forty primiparous women satisfying specific risk criteria participated in a longitudinal study with their children from pregnancy until 18 month of child age. A multiple psychological and socioeconomic assessment was performed. The Emotional Availability Scales were used to measure the quality of emotional exchanges between mother and child at 12 months and the Attachment Q-Sort served as a measure of child attachment security at 18 months. Results highlight both the effect of specific single factors, considered at a continuous level, and the cumulative risk effect of different co-occurring factors, considered at binary level, on mother–child interaction quality and child attachment security. Implication for the selection of inclusion criteria of intervention programs that support parenting skills in at-risk families are discussed.

## INTRODUCTION

Child physical and psychological development is largely influenced by parent–child relationship (Bornstein, 2002). Through their caregiving, parents supply their children with the experiences and support they need for achieving their developmental milestones (Brinker et al., 1994). An extensive body of research on human parenting has provided evidence that quality of caregiving is essential in determining the level of children’s social emotional as well as cognitive development (Bornstein and Tamis-LeMonda, 1989; Landry et al., 2006; Dexter et al., 2013). Specifically, sensitive and responsive parenting promotes a secure attachment relationship (De Wolff and van IJzendoorn, 1997; Nievar and Becker, 2008), which in turn is predictively associated with positive outcomes in numerous domains of child development (Bar-Haim et al., 2000; Vondra et al., 2001; Belsky and Pasco Fearon, 2002; Bernier and Meins, 2008; Dexter et al., 2013). However, the parent–child relationship can be affected by several psychosocial and socio-demographic risk factors that undermine its quality and in turn play a negative role in short and long term child psychological wellbeing (Sameroff, 1998, 2000; Choe et al., 2013). Classical research on the psychological development of children living in at risk families supports additive models according to which psychological problems are not the result of one specific risk factor but may be instead predicted by the combined presence of different factors (Greenberg et al., 1993; Lyons-Ruth et al., 1993; Hubbs-Tait et al., 1996). Also, established models of parenting (Belsky, 1984) postulate that the quality of the parent–child relationship is the integrated result of three sets of factors: parent characteristics, infant characteristics, and context which can influence parenting in a supportive or stressful way. Accordingly, many studies have investigated to which extent attachment security can be explained by theoretically relevant risk factors, such as low family socio-economic status (SES), maternal depression and other psychological symptoms, young maternal age, single parenting. However, most studies concentrated on one specific risk factor at the time, or several co-occurring factors that could not be disentangled. The aim of the present study was to examine the influence on dyadic emotional availability (EA; Biringen and Robinson, 1991; Biringen, 2000), as an index of mother–child interaction quality, and on child attachment security of different psychosocial and socio-demographic risk factors in at-risk families living in Northern Italy. Results in this direction may guide the selection of inclusion criteria for effective intervention programs that promote sensitive and responsive parenting in at-risk families.

### ATTACHMENT SECURITY AND EMOTIONAL AVAILABILITY

Secure attachment has been shown to positively influence child social emotional as well as cognitive adaptation beyond infancy (Bar-Haim et al., 2000; Vondra et al., 2001; Dexter et al., 2013). Secure mother–child attachment is the result of adequate maternal sensitivity, as postulated by Ainsworth et al. (1978) and demonstrated by a strong body of literature (NICHD Early Child Care Research Network, 1997, 2006). However, especially in high risk populations, the strength of this association may be modest and sensitivity may just partially explain child attachment security (Ward and Carlson, 1995; Seifer et al., 1996). Indeed, different risk factors can affect maternal sensitivity and child attachment security in many ways, complicating their associations. When studying the impact of risk factors on mother–child relationship, it may be appropriate to focus on more complex constructs within the theoretical attachment framework that expands that of maternal sensitivity. In this perspective, EA is a crucial construct (Biringen and Robinson, 1991; Aviezer et al., 1999; Bretherton, 2000; EA; Biringen and Robinson, 1991; Biringen, 2000) is a relationship construct based on attachment theory (Ainsworth et al., 1978) integrated with Emde’s (1980) emotions perspective. It refers to the quality of emotional exchanges between parents and their children, focusing on the two partners’ accessibility to each other and their ability to read and respond appropriately to one another’s communications (Biringen and Robinson, 1991). Studies of EA suggest that the construct not only plays a role in the prediction of attachment (Easterbrooks and Biringen, 2000, 2005) but also show significant and meaningful associations with many discrete affective indices of parent–child interaction (Robinson et al., 1993; Zimmerman and McDonald, 1995; Kogan and Carter, 1996), and can be considered a global index of the overall quality of the parent–child affective relationship (Biringen, 2000).

### PSYCHOSOCIAL RISK FACTORS FOR PARENT–CHILD RELATIONSHIP

Among the most investigated factors thought to have a detrimental effect on parent–child relationship and child healthy development are low SES of the family and maternal psychopathology. SES influences physical and psychological health as well as the chances of social and cultural achievements during the all life span (Ensminger and Fothergill, 2003). Children born and raised in low SES families have higher chances of perinatal negative outcomes and fewer chances of receiving the appropriate physical cares and cognitive stimulation they need for their healthy development (Yoshikawa et al., 2012). Low parental SES has been predictively associated with lower children IQ and school achievements (Bradley and Corwyn, 2002; Gottfried et al., 2003; Clearfield and Niman, 2012). The effect of low SES on child development is partially mediated by reduced parenting quality (McLoyd and Wilson, 1991; Fish, 2001). Parenting quality is thought to be affected by the increased instability and stresses connected to low income and poor education conditions (Bronfenbrenner, 1979; Aber et al., 2000; Bradley and Corwyn, 2002; Lemelin et al., 2006; Seow, 2012). Low SES mothers are reported to show lower levels of synchronic and responsive behavior when interacting with their children (Tamis-LeMonda et al., 2001; Ammaniti et al., 2002; Steele et al., 2002; van Bakel and Riksen-Walraven, 2002; Aviezer et al., 2003; Evans et al., 2005; Tarabulsy et al., 2005; Shin et al., 2006) as well as more inconsistent, punitive, and coercive parenting (McLoyd and Wilson, 1991). Lower levels of attachment security and a higher rate of disorganized attachment have been clearly documented in studies on low SES and low income families (Lyons-Ruth et al., 1991; NICHD Early Child Care Research Network, 1997; van IJzendoorn et al., 1999). However, some level of inconsistency exists in this body of research and meta-analytic studies demonstrated comparable levels of attachment security between low SES and middle class children when isolated cases of neglect and maltreatment where excluded (Spieker and Booth, 1988).

A plethora of research has documented negative associations between maternal psychopathology and child psychological wellbeing (Downey and Coyne, 1990; Cummings and Davies, 1994; Seifer and Dickstein, 2000; Burstein et al., 2012). This field of research has been dominated by studies on maternal depression showing its detrimental effect on mother–child relationship and child healthy development (Downey and Coyne, 1990; Campbell et al., 1992, 1995; Beck, 1995; Murray et al., 1999; Edhborg et al., 2001; Nagata et al., 2004; Toth et al., 2009; McCabe, 2014). Maternal depression has been empirically linked to lower levels of attachment security (Lyons-Ruth et al., 1990; Lyons-Ruth et al., 1990; Murray, 1992; Teti et al., 1995; Goodman and Gotlib, 1999, 2002; Lyons- Martins and Gaffan, 2000) and diminished maternal sensitivity connected to the nature of depressive symptoms has been accounted to mediate this association (Ammaniti et al., 2007). However, the strength of this association has been found to be modest and maternal depression alone may not result in inadequate parenting quality or attachment insecurity for some children (van IJzendoorn et al., 1999; Atkinson et al., 2000; Toth et al., 2009).

### SOCIO-DEMOGRAPHIC RISK FACTORS FOR PARENT–CHILD RELATIONSHIP

Other conditions that have been investigated as potential risk factors in the parenting literature are two socio-demographic factors, namely young maternal age, and, to a lower extent, single parenting. However, both factors are thought to affect parent–child relationship mainly when occurring together with psychosocial risk conditions, as those described above, which indeed are especially frequent among young and single mothers (Rosenkrantz Aronson and Huston, 2004; Letourneau et al., 2004; Svoboda et al., 2012).

Adverse health and psychological outcomes have been associated with adolescent pregnancy and parenting (Long, 2009) including gestational hypertension, preterm labor and delivery (Sieger and Renk, 2007), and children’s cognitive and language delayed development (Keown et al., 2001; Rafferty et al., 2011). Being themselves in a process of “separation-individuation” and in a period of emotional and social changes during the transition to adulthood, adolescent mothers may find it difficult to face the challenges of parenthood. There is a strong body of research showing that compared to older mothers, adolescent mothers display less desirable childrearing attitudes, lower sensitivity and diminished (EA; Pomerleau et al., 2003; Easterbrooks et al., 2005; Bornstein and Putnick, 2007). Accordingly, researchers found that children of adolescent mothers are less likely to develop a secure attachment and more likely to develop behavioral problems (Madigan et al., 2006; Moran et al., 2008).

Raising a child without the help of a partner exposes the mothers to more challenges, stress and fatigue, leading to higher chances of psychological problems (Broussard et al., 2012). Children raised only by the mother show poorer outcomes in several areas of development. Research in this domain has demonstrated lower levels of social emotional adaptation, social competence, cognitive scores, school achievements as well as higher rates of anxiety disorders and deviant behaviors in children raised in single-mother compared to two-parents families (Carlson and Corcoran, 2001; Weinraub et al., 2002; Tremblay and Japel, 2003; Fergusson et al., 2007; Scharte and Bolte, 2011; Shaw et al., 2012). Studies on attachment relationship between single mothers and their children showed inconsistent results ranging from increased (Golombok et al., 1997) to slightly decreased children’s attachment security (Rosenkrantz Aronson and Huston, 2004).

All the mentioned risk factors may negatively affect mother–child relationship quality and children’s attachment security eventually affecting child healthy development. However, the predictive value of each risk factor appears to be moderated by the co-occurrence of other risk factors in a complex additive model. Nevertheless most of the studies within this body of literature took into account one risk factor at the time. Moreover, some inconsistency in the results might be related to the tendency to operationalize risk factors only in a dichotomic way by comparing families with and without a specific risk condition, disregarding factors’ intensity that can be instead accounted by considering risk factors at a continuous level (McCabe, 2014). Starting from the above considerations, the present study aimed to assess the influence of psychosocial and socio-demographic risk factors on mother–child EA and on children’s attachment security in a sample of at-risk families living in Northern Italy. In an attempt to identify the specific effect on mother–child relationship of each risk factor as well as the cumulative effect of their co-occurrence, low family SES, maternal psychopathology, maternal young age, and single parenting, were analyzed both as dichotomic and as continuous variables in a longitudinal design. We specifically hypothesized to find modest negative associations between continuous measures of risk factors and the EA and attachment measures. Also, we didn’t expect differences in EA and attachment security measures between groups of dyads with or without single risk factors. Finally, we expected that EA and attachment security measures would be higher in dyads presenting socio-demographic factors alone compared to dyads presenting psychosocial risk factors alone or a combination of both. Results of this explorative study would be crucial for the identification of the families at higher risk for low relationship quality and attachment security who might profit of prevention intervention that promote emotionally available mother–child relationship and in turn reduce the risk of poor developmental outcomes.

## MATERIALS AND METHODS

### PARTICIPANTS

This study involved 40 first-time mothers with specific psychosocial and/or socio-demographic risk factors and their healthy full term children living in three different areas (A, B, and C) of Northern Italy. At the time of recruitment mothers had a mean age of 27.32 years (SD = 6.56) and they were in the third trimester of pregnancy. Measures for the present study were collected when children were aged 3, 6, 12, and 18 months (with a 2-weeks flexibility interval). Participants were part of a broader intervention study on at-risk families in either the intervention or the control group (group assignment was controlled as appropriate in the analyses for the purposes of the present study, see “Materials and Methods” section). Participation in the project was proposed by midwifes, OB/GYN doctors and psychologists of the local public health centers who received specific information about the project and the inclusion criteria. After consensus was obtained, mothers were visited by a trained psychologist to verify through clinical interview and psychological assessment that at least one of the following inclusion criteria was satisfied: (1) age under 22 years; (2) single mother; (3) low family SES [family income under 13.125,90 euros (poverty threshold according to ISTAT for 2011); less than 10 years of education]; (3) high indexes of psychopathological symptoms assessed through standardized questionnaires [Global Severity Index, GSI >1 at the Symptoms-Checklist 90-R, (SCL 90-R; Derogatis, 1994 ) and/or Edimburg Postnatal Depression Scale (EPDS; Cox et al., 1987) score >9] or being actually followed by the local public mental health services. Twenty-three mothers (57,5%) satisfied criteria for only one of the risk factors: 15 for psychopathological symptoms (37,5%), 3 for single parenting (7,5%), 3 for low family SES 3 (7,5%), and 2 for young age (5,0%). In the remaining sample, 14 mothers (35,0%) satisfied criteria for two risk factors, and 3 (7,5%) for 3 risk factors. Eighty-seven percent of the contacted mothers accepted to participate in the study and drop out was 12%. Participants gave written informed consent for their participation in the study. The study protocol was approved by the local ethical committee.

### PROCEDURE

The protocol of the broader longitudinal study in which the participants were involved included several home visits of a trained psychologist for test administration, mother–child interaction observation and video recording. Measures of maternal risk factors used for the present study were collected during home visits at pregnancy and at child age three and six Measures of mother–child relationship quality and child attachment security were taken during home visits at ages 12 and 18 months, respectively.

#### Measures of psychosocial and socio-demographic risk factors

Maternal risk factors were considered both at a dychotomic and at a continuous level. (A) Dychotomic classifications of the mothers for low family SES, maternal psychopathology, young age and single parenting are described in the *Participants* section where the inclusion criteria are defined. (B) As continuous measures of psychosocial risk factors the following questionnaire scores were considered: the Four-Factor Index of Social Status (Hollingshead, unpublished manuscript) as a measure of family SES; the GSI of the SCL 90-R (Derogatis, 1994) and the EPDS (Cox et al., 1987) score as measures of maternal psychopathological symptoms.

#### Emotional availability measure

Emotional availability in a subsample of 25 mother–child dyads was evaluated from 15-min of free-play at home interaction videorecorded continuously by a female filmmaker. A standard set of age-appropriate toys was used and mothers were instructed to play with their children in their usual way, disregarding the observer presence. Observations were coded using the emotional availability scales (EAS): Infancy to Early Childhood Version (EAS 4th Edn; Biringen, 2008). These Scales consist of six dimensions concerned with emotional regulation in the parent–child dyad. Four dimensions address the EA of the parent in relation to the child (sensitivity, structuring, non-intrusiveness, and non-hostility), and two address the EA of the child in relation to the parent (responsiveness and involving). All scales range from 1 (highly emotional unavailable) to 7 (highly emotional available) points and scores are given based on seven subcategories. The Sensitivity scale assesses the parent’s appropriate affectivity, acceptance, flexibility, clarity of perceptions, affect regulation, and variety and creativity in play displayed toward the child. Structuring assesses the degree to which the parent provides rules, regulations, and a supportive framework for interaction while appropriately scaffolding child play, exploration, or routine. Non-intrusiveness refers to the ability to be available for the child without being over-directive, over-stimulating, or overprotective. Non-hostility assesses the degree of hostility both in covert and overt forms. Responsiveness focuses on the child’s interest in exploring on his or her own and in responding to the mother’s bids (i.e., the balance between connection and autonomy) as well as on the extent of the child’s enjoyment of the interaction. Involving of mother assesses the child’s ability and willingness to engage the mother in interaction. The flexible nature of the Scales, which can be used with children from infancy to early childhood, and the choice of an ecological context of free-play with a standard toy set for pre-school children allowed us to use the same observational situation for all participants. Coding was carried out by two independent coders who were first trained on the EAS to obtain satisfactory interrater reliability with the codings the author of the EAS 4th edition and then between themselves. Interrater reliability [Biringen, 2005 was assessed using average absolute agreement intraclass correlation coefficients (ICC); McGraw and Wong, 1996] on 15% of the interactions coded, and ICCs ranged from 0.81 to 0.93.

#### Attachment security measure

Child attachment security was measured using the Italian version of the Attachment Q-Sort (AQS; Weters, 1987; Cassibba and D’Odorico, 2009) applied at 18 months of child age by a trained observer after 2–4 home visits lasting ca 1,5 h each. AQS is a Q-Sort method to evaluate child security in relation to the quality of child secure-base behavior (Vaughn and Waters, 1990). The Italian version includes 90 items describing child behavior that the observer has to sort into nine piles according to similarity with the infant’s behavior (Vaughn and Waters, 1990; Cassibba et al., 2000). By comparing this sorting with a composite description of the “hypothetically most secure infant” provided by experts in the field of attachment theory, a score for attachment security is obtained (Cassibba et al., 2000) that is thought to reflect the degree of similarity of the target child to the prototypical securely attached child (Teti and Ablard, 1989; Bretherton et al., 1989; Howes and Hamilton, 1992).

## RESULTS

### PRELIMINARY ANALYSIS AND ANALYTIC PLAN

Distributions of the dependent variables were examined for normalcy which was confirmed for all EAS and for AQS scores. As the EAS and the AQS are measures of theoretically associated constructs, bivariate correlation analysis was performed between AQS score and EAS scores in order to verify that these measures were in fact empirically associated in this study. Results, highlighting positive association between the AQS and two scales of the EAS: mother non-intrusiveness [*r*(36) = 0.38, *p* < 0.05], and child responsiveness [*r*(36) = 0.40, *p* < 0.05], constitute relevant confirmation of the validity of the instruments used to measure the dependent variables.

Bivariate correlation analyses were performed to check associations of AQS and EAS scores with the risk factors measured as continuous variables. Then, risk factors were considered in binary fashion and for each of them (young maternal age, single parenting, maternal psychopathology, family SES), separate ANCOVAs were performed with AQS and EAS scores as dependent variables, and each risk factor (present or absent) as between factor. Finally, the risk factors were aggregated into a three-level between factor (socio-demographic only, psychosocial only, both) which served as independent variable in further separate ANCOVAs with AQS and EAS scores as dependent variables. Participants were part of a broader intervention study either in the target or control group; as a precaution, group assignment was included as covariate in all the analyses of variance described above.

### DESCRIPTIVE STATISTICS

**Table 1** show the means and standard deviations of AQS and EAS scores for dyads with and without each risk factor and in the whole sample. **Table 2** shows descriptive statistics of AQS and EAS scores for dyads with socio-demographic risk factors alone, psychosocial risk factors alone, and with both kind of risk factors.

**Table 1:** Attachment Q-Sort and Emotional Availability Scales.

	Total Sample		Young maternal age				Single parenting				Psychopathological symptoms				Low family SES			
	(*N* = 40)		Presence (*N* = 12)		Absence (*N* = 28)		Presence (*N* = 13)		Absence (*N* = 27)		Presence (*N* = 23)		Absence (*N* = 17)		Presence (*N* = 12)		Absence (*N* = 28)	
	*M*	*SD*	*M*	*SD*	*M*	*SD*	*M*	*SD*	*M*	*SD*	*M*	*SD*	*M*	*SD*	*M*	*SD*	*M*	*SD*
Attachment Q-Sort	0.27	0.38	0.36	0.26	0.26	0.39	0.35	0.42	0.26	0.33	0.25	0.33	0.34	0.39	0.15	0.41	0.35	0.33
Sensitivity}	5.10	0.97	5.00	0.87	5.29	0.98	5.44	0.98	5.10	0.93	5.29	0.97	5.05	0.91	5.06	1.07	5.26	0.89
Structuring	4.82	1.15	4.61	1.41	5.18	0.89	5.25	0.71	4.90	1.21	5.15	0.93	4.77	1.31	4.61	1.34	5.18	0.93
Non-intrusiveness	5.17	0.80	5.22	0.51	5.34	1.00	5.13	0.92	5.38	0.86	5.50	0.88	5.00	0.78	5.11	0.93	5.39	0.84
Non-hostility	5.69	0.89	5.56	0.85	5.82	1.03	5.94	1.12	5.65	0.92	5.76	1.00	5.68	0.96	5.83	1.03	5.68	0.96
Responsiveness	5.15	0.93	5.28	0.62	5.26	1.14	5.25	0.93	5.28	1.03	5.35	1.07	5.14	.87	5.17	0.94	5.32	1.03
Involving	4.88	1.20	4.89	0.74	5.11	1.24	5.00	1.23	5.05	1.08	5.18	1.19	4.82	0.96	5.06	1.18	5.03	1.09

*Scores in all dyads and for each risk factor: young age, single parenting, psychopathological symptoms, and low family SES.*

**Table 2:** Descriptive and inferential statistics for Attachment Q-Sort and Emotional Availability Scales scores for type of risk factors.

	Socio-demographic risk only (*N* = 10)		Psychosocial risk only (*N* = 19)		Both risk factors (*N* = 11)			
	*M*	*SD*	*M*	*SD*	*M*	*SD*	*F(2,37)*	*p*
Attachment Q-Sort	0.35	0.40	0.24	0.38	0.06	0.37	3.26	0.03
Sensitivity	5.42	0.86	5.21	0.89	5.00	1.13	1.46	0.25
Structuring	5.33	0.88	5.18	0.91	4.44	1.40	2.09	0.13
Non-intrusiveness	5.42	0.59	5.39	0.96	5.06	0.90	1.71	0.19
Non-hostility	5.92	0.97	5.75	0.92	5.56	1.15	1.30	0.30
Responsiveness	5.58	0.67	5.21	1.16	5.13	0.92	0.94	0.44
Involving	5.17	0.75	5.07	1.19	4.88	1.25	1.39	0.27

### CORRELATIONAL ANALYSIS

Results showed a positive correlation of mother’s sensitivity [*r*(36) = 0.47, *p* < 0.01], structuring [*r*(36) = 0.55, *p* < 0.001], and non-hostility [*r*(36) = 0.38, *p* < 0.05) with family SES. Correlation between family SES and AQS scores was within the moderate range but did not reach significance [*r*(40) = 0.33, *p* = 0.09]. AQS and EAS scores were not significantly associated with measures of maternal psychopathology, i.e., SCL 90-R GSI and EPDS scores (**Table 3**).

**Table 3:** Bivariate correlations of AQS and Emotional Availability Scales scores with family SES, maternal age, SCL 90-R (GSI), EPDS.

	AQS	Sens.	Structure	Non-intr.	Non-host.	Resp.	Invol.
Age	-0.10	0.33	0.50**	0.25	0.35*	0.19	0.30
SES	0.33	0.47**	0.55**	0.30	0.38*	0.29	0.30
SCL 90-R	0.12	0.09	0.11	0.00	-0.08	-0.05	-0.22
EPDS	-0.13	-0 .16	-0.25	-0.04	-0.08	-0.06	-0.05

Significance: *p < 0.05, **p < 0.01. Sens(itivity); Struct(uring); Non-intr(usiveness); Non-host(ility); Resp(onsiveness); Invol(ving).

### GROUP COMPARISONS

No significant differences were found in AQS and EAS scores of dyads differentiated according to the presence/absence of each of the risk factors as defined for the inclusion criteria. However, dyads presenting a low family SES had lower scores of maternal structuring and non-intrusiveness scores [*F*(1,34) = 3.13, *p* < 0.062 and *F*(1,34) = 2.98, *p* < 0.07, respectively], compared to dyads who did not meet the criteria for this risk factor, but the results did not reach statistical significance.

A significant difference was found between dyads with different type of risk factors (socio-demographic only, psychosocial only, both), *F*(2,37) = 3.26, *p* < 0.05, η^2^ = 0.31. *Post hoc* test showed that attachment security was higher in dyads with demographic risk factor only compared to dyads with both demographic and socio-psychological fragilities, whereas attachment security of children in dyads presenting only psychosocial risk factor did not differ significantly from the other two groups. (*p* < 0.02, Bonferroni Correction for multiple comparisons; **Table 2**).

## DISCUSSION

Among several influential variables, parents can be considered the “final common pathway” for children development, growth and adaptation (Bornstein, 2002). Specifically, the quality of mother–child interaction and attachment are key determinants of child social emotional adaptation and cognitive development (Bowlby, 1969; Ainsworth et al., 1978; Belsky and Pasco Fearon, 2002; Bernier and Meins, 2008). Different risk factors can affect mother–child relationship resulting in negative children outcomes as demonstrated by a strong body of research (Sameroff, 1998; Choe et al., 2013). Psychosocial risk factors, such as low family SES and maternal psycopathology, and socio-demographic risk factors, such as very young maternal age and single parenting, are among the most theoretically relevant and empirically investigated risk conditions. Although results of this line of research are substantially coherent in identifying the detrimental effect of each factor, they also point out to a complex cumulative model of risk according to which a single factor may not be influential itself whereas its predictive value might be moderated by the association with other risk conditions or by its degree of intensity (Belsky, 1984; Greenberg et al., 1993; Lyons-Ruth et al., 1993; Hubbs-Tait et al., 1996). Several intervention programs have been developed for at-risk families and some have shown positive effect on mother–child relationship quality (Bakermans-Kranenburg et al., 2003) reducing the impact of risk factors on mother and child psychological health (Kohlhoff and Barnett, 2013). Starting from the above considerations, this study aimed at investigating the effect of psychosocial and socio-demographic risk factors on mother-child EA, measured with the EAS at 12 months of child age and child attachment security, measured with the AQS at 18 months of child age, in at risk dyads living in Northern Italy. Specifically, we focused on low family SES, maternal psycopathology, young maternal age and single parenting considered both at a continuous (where appropriate) and at a dichotomic level in order to investigate the contribution of each factor as well as the potential additive effect of more factors on mother–child relationship measures. We had a set of specific hypotheses: we expected 1) that intensity of each risk condition would show modest negative association with mother–child EA and child attachment security. Also, we hypothesized 2) to find no differences in our dependent variables between dyads differentiated according to the presence/absence of each of the risk factor, but we expected 3) socio-demographic risk factors to show a smaller effect on EA and child attachment security then psychosocial risk factors or the combination of the two type of fragility. Preliminary analyses of the selected measures validity verified positive association between some maternal and child EA scales and AQS score.

With respect to our first hypothesis, we found that in our sample the level of family SES was significantly positively associated with different dimensions of maternal EA, namely sensitivity, structuring and non-hostility. Mothers with a higher socioeconomic level recognize and respond more appropriately to child signals, structure the interaction in a way that better foster child play and exploration and are more able to limit their display of negative emotions during interaction. The positive association between SES and the quality of mother interactive behavior measured in similar and different ways has been observed in previous studies (Rosen et al., 2003; Aviezer et al., 2003; Evans et al., 2005; Tarabulsy et al., 2005) and attributed to higher cognitive, emotional, material, and social resources that characterize high compared to low SES families. The association between family SES and child attachment security at 18 months did not reach significance but the coefficient was in the moderate range and the small *n* of our sample may have reduced the power of our analyses. Such association has been reported in previous studies (Lyons-Ruth et al., 1991; NICHD Early Child Care Research Network, 1997; van IJzendoorn et al., 1999) with some notable exceptions (Spieker and Booth, 1988). We also found maternal age to be positively associated with mother EA and specifically to mother structuring and non-hostility, in line with previous studies demonstrating that compared to older mothers, younger mothers show more limited knowledge about childrearing and child development, lower confidence in their parenting skills (Bornstein and Putnick, 2007) and a less emotionally available interactive style (Pomerleau et al., 2003; Gazzotti et al., 2010). Instead, no association was found between maternal age and child attachment security. Surprisingly, our measures of maternal psychopathology, i.e., SCL-90-R and EPDS (at 3 and 6 months of age respectively), showed no associations with either mother–child EA and child attachment security. Yet, psychopathological symptoms of the mother, and depression in particular, have been repeatedly reported to predict negative outcomes terms of maternal sensitivity and child attachment security though highlighting only moderate association (van IJzendoorn et al., 1999; Atkinson et al., 2000). Our findings might been explained by the small inter-subject variability in questionnaire scores of a sample including only mothers at risk for socio-demographic and/or psychosocial risk factors.

As expected, according to our second hypotheses, in our sample of at-risk mother-child dyads we did not find any effect of the single risk factors considered in a binary fashion, i.e., in terms of presence or absence, on either EA and attachment security, with the exception of the tendency of mothers with low SES to display lower structuring and non-intrusiveness which, however, did not reach significance. With respect to our third hypothesis, when we explored the effect of the type of risk factors by aggregating them into a three level variable, i.e., comparing dyads with socio-demographic risk factors only (young maternal age and/or single parenting), those with psychosocial risk factors only (low family SES and/or maternal psychopathology) and those who had both type of risk factors, results highlighted higher attachment security in children of mothers with socio-demographic risk factors only compared to those with both kinds of risk conditions, with attachment security level of children with psychosocial risk factors only somehow in the middle. Although, AQS does not allow to classify secure vs. non-secure children, when looking at previous studies, it becomes evident that mean scores of children in dyads with socio-demographic risk only are surprisingly similar to those of the no-risk typical sample used in a study of AQS reliability and validity in the Italian population (Cassibba et al., 2000). Altogether these findings speak in favor of classical approaches to psychological development of children living in at-risk families according to which the predictive value of each factor is moderated by its intensity and by the co-occurrence of other specific risk conditions (Belsky, 1984; Greenberg et al., 1993; Lyons-Ruth et al., 1993). Also, these findings are consistent with theoretical models (Belsky, 1984) and psychometrics evidences (Markon et al., 2011) according to which considering risk variables in a binary, instead of continuous, fashion might lower the possibility to describe the risk conditions in a valid and complete way (McCabe, 2014).

A crucial limitation of the present study concerns the sample size and the small *n*s of the subgroups of dyads satisfying each selected risk criterion or a specific association of criteria; a wider sample would allow more sophisticated and informative analyses to identify the contributing role of each single risk factors as well as their reciprocal additive effects. Moreover, the inclusion of other theoretically relevant variables, such as child temperament and maternal attachment security would enrich the study in terms of deepening our understanding of the effects of the selected risk factors on mother–child relationship and attachment. Nevertheless, the results of this study may have some important implications for the selection of inclusion criteria for prevention programs that promote emotionally available caregiving and secure attachment in at-risk families, at least within the target geographical area. In our sample, dyads with co-occurring socio-demographic and psychosocial risk factors show the lowest level of attachment security suggesting that they might be the most urgent targets for prevention intervention programs, whereas dyads with socio-demographic risk factors alone appear to be less in need of such intervention. Moreover, looking at risk factor intensity, we found that family SES and maternal age were positively associated with maternal EA, suggesting that mother child-dyads displaying low family SES or very young maternal age might deserve special attention for the risk of emotionally unavailable and undesirable interactive style.

## Conflict of Interest Statement

The authors declare that the research was conducted in the absence of any commercial or financial relationships that could be construed as a potential conflict of interest.

## References

[B1] Aber J. L. Jones S. Cohen J. (2000). “The impact of poverty on the mental health and development of very young children” in *Handbook of Infant Mental Health,* ed. Zeanah C. H. (New York: Guilford Press) 113–128

[B2] Ainsworth M. D. S. Blehar M. C. Waters E. Wall S. (1978). *Patterns of Attachment: A Psychological Study of the Strange Situation*. Hillsdale, NJ: Lawrence Erlbaum

[B3] Ammaniti M. Cimino S. Trentini C. (2007). *Quando le Madri Non Sono Felici. La Depressione Post-Partum*. Roma: Il Pensiero Scientifico Editore

[B4] Ammaniti M. Sergi G. Speranza A. M. Tambelli R. Vismara L. (2002). Maternità a rischio, interazioni precoci e attaccamento infantile. *Età Evol.* 72 61–67

[B5] Atkinson L. Paglia A. Coolbear J. Niccols A. Parker K. C. Guger S. (2000). Attachment security: a meta-analysis of maternal mental health correlates. *Clin. Psychol. Rev.* 20 1019–1040 10.1016/S0272-7358(99)00023-9 11098398

[B6] Aviezer O. Sagi A. Joels T. Ziv Y. (1999). Emotional availability and attachment representations in kibbutz infants and their mothers. *Dev. Psychol.* 35 811–821 10.1037/0012-1649.35.3.811 10380871

[B7] Aviezer O. Sagi-Schwartz A. Koren-Karie N. (2003). Ecological constraints on the formation of infant-mother attachment relations: when maternal sensitivity becomes ineffective. *Infant Behav. Dev.* 26 285–299 10.1016/S0163-6383(03)00032-8

[B8] Bakermans-Kranenburg M. J. van IJzendoorn M. H. Juffer F. (2003). Less is more: meta-analyses of sensitivity and attachment interventions in early childhood. *Psychol. Bull.* 129 195–215 10.1037/0033-2909.129.2.195 12696839

[B9] Bar-Haim Y. Sutton D. B. Fox N. A. Marvin R. S. (2000). Stability and change of attachment at 14 24 and 58 months of age: behavior, representation, and life events. *J. Child Psychol. Psychiatry* 41 381–388 10.1111/1469-7610.00622 10784085

[B10] Beck C. H. (1995). The effects of postpartum depression on maternal–infant interaction: meta-analysis. *Nurs. Res.* 44 298–304 10.1097/00006199-199509000-00007 7567486

[B11] Belsky J. (1984). The determinants of parenting: a process model. *Child Dev.* 55 83–96 10.2307/1129836 6705636

[B12] Belsky J. Pasco Fearon R. M. (2002). Infant-mother attachment security, contextual risk, and early development: a moderational analysis. *Dev. Psychopathol.* 14 293–310 10.1017/S0954579402002067 12030693

[B13] Bernier A. Meins E. (2008). A threshold approach to understanding the origins of attachment disorganization. *Dev. Psychol.* 44 969–982 10.1037/0012-1649.44.4.969 18605828

[B14] Biringen Z. (2000). Emotional availability: conceptualization and research findings. *Am. J. Orthopsychiatry* 70 104–114 10.1037/h0087711 10702855

[B15] Biringen Z. (2005). Training and reliability issues with the emotional availability scales. *Infant Ment. Health J.* 26 404–405 10.1002/imhj.20060 28682446

[B16] Biringen Z. (2008). *Emotional Avalilability Scales,* IV Edn Unpublished manual, Department of Human Development and Family Studies, Colorado State University Fort Collins, CO

[B17] Biringen Z. Robinson J. (1991). Emotional availability in mother-child interactions: a reconceptualization for research. *Am. J. Orthopsychiatry* 61 258–271 10.1037/h0079238 2048641

[B18] Bornstein M. H. Putnick D. L. (2007). Chronological age, cognitions, and practices in European American mothers: a multivariate study of parenting. *Dev. Psychol.* 43 850–864 10.1037/0012-1649.43.4.850 17605519 PMC5827928

[B19] Bornstein M. Tamis-LeMonda C. S. (1989). “Maternal responsiveness and cognitive development in children” in *Maternal Responsiveness: Characteristics and Consequences* ed. Bornstein M. H. (San Francisco, CA: Jossey-Bass) 49–61 10.1002/cd.23219894306 2710392

[B20] Bornstein M. H. (2002). “Parenting infants,” in *Handbook of Parenting, Vol. 1 Children and Parenting* ed. Bornstein M. H. (Mahwah, NJ: Lawrence Erlbaum Associates) 3–43

[B21] Bowlby J. (1969). *Attachment and Loss: Attachment* Vol 1 London: Hogarth Press

[B22] Bradley R. H. Corwyn R. F. (2002). Socioeconomic status and child development. *Annu. Rev. Psychol.* 53 371–399 10.1146/annurev.psych.53.100901.135233 11752490

[B23] Bretherton I. (2000). Emotional availability: an attachment perspective. *Attach. Hum. Dev.* 2 233–241 10.1080/14616730050085581 11707913

[B24] Bretherton I. Biringen Z. Ridgeway D. Maslin C. Sherman M. (1989). Attachment: the parental perspective. *Infant Ment. Health J.* 10 203–221 10.1002/1097-0355(198923)10:3<203::AID-IMHJ2280100307>3.0.CO;2-8

[B25] Brinker R. P. Seifer R. Sameroff A. J. (1994). Relations among maternal stress, cognitive development, and early intervention in middle- and low-SES infants with developmental disabilities. *Am. J. Mental Retard.* 98 463–480 8148123

[B26] Bronfenbrenner U. (1979). *The Ecology of Human Development*. Cambridge, MA: Harvard University Press

[B27] Broussard C. A. Joseph A. L. Thompson M. (2012). Stressors and coping strategies used by single mothers living in poverty. *Affilia* 27 190–204 10.1177/0886109912443884

[B28] Burstein M. Stanger C. Dumenci L. (2012). Relations between parent psychopathology, family functioning, and adolescent problems in substance-abusing families: disaggregating the effects of parent gender. *Child Psychiatry Hum. Dev.* 44 631–647 10.1007/s10578-012-0288-z 22392413 PMC4383170

[B29] Campbell S. B. Cohn J. F. Meyers T. (1995). Depression in first time mothers: mother-infant interaction and depression chronicity. *Dev. Psychol.* 31 349–357 10.1037/0012-1649.31.3.349

[B30] Campbell S. B. Cohn J. F. Flanagan C. Popper S. Meyers T. (1992). Course and correlates of postpartum depression during the transition to parenthood. *Dev. Psychopathol.* 4 29–47 10.1017/S095457940000554X

[B31] Carlson M. J. Corcoran M. E. (2001). Family structure and children’s behavioral and cognitive uutcomes. *J. Marriage Fam.* 63 779–792 10.1111/j.1741-3737.2001.00779.x

[B32] Cassibba R. D’Odorico L. (2009). *La Valutazione Dell’Attaccamento Nella Prima Infanzia. L’Adattamento Italiano Dell’Attachment Q-Sort (AQS) di Everett Waters*. Milano: Franco Angeli

[B33] Cassibba R. van IJzendoorn M. H. D’Odorico L. (2000). Attachment and play in child care centers: reliability and validity of the attachment Q-sort for mothers and professional caregivers in Italy. *Int. J. Behav. Dev.* 24 241–255 10.1080/016502500383377

[B34] Choe D. E. Olson S. L. Sameroff A. J. (2013). Effects of early maternal distress and parenting on the development of children’s self-regulation and externalizing behaviour. *Dev. Psychopathol.* 25 2 437–453 10.1017/S0954579412001162 23627955

[B35] Clearfield M. W. Niman L. C. (2012). SES affects infant cognitive flexibility. *Infant Behav. Dev.* 35 29–35 10.1016/j.infbeh.2011.09.007 22018718

[B36] Cox J. L. Holden J. M. Sagovsky R. (1987). Detection of postnatal depression. Development of the 10-item Edinburgh Postnatal Depression Scale. *Br. J. Psychiatry* 150 782–786 10.1192/bjp.150.6.782 3651732

[B37] Cummings E. M. Davies P. T. (1994). Maternal depression and child development. *J. Child Psychol. Psychiatry* 35 73–112 10.1111/j.1469-7610.1994.tb01133.x 8163630

[B38] De Wolff M. S. van IJzendoorn M. H. (1997). Sensitivity and attachment: a meta-analysis on parental antecedents of infant attachment. *Child Dev.* 68 571–591 10.1111/j.1467-8624.1997.tb04218.x 9306636

[B39] Derogatis L. R. (1994). *Symptom Checklist-90-R: Administration, Scoring, and Procedures Manual* 3rd edn. Minneapolis, MN: National Computer Systems

[B40] Dexter C. A. Wong K. Stacks A. M. Beeghly M. Barnett D. (2013). Parenting and attachment among low-income African American and Caucasian preschoolers. *J. Fam. Psychol.* 27 629–38 10.1037/a0033341 23795606

[B41] Downey G. Coyne J. C. (1990). Children of depressed parents. *Psychol. Bull.* 108 50–76 10.1037/0033-2909.108.1.50 2200073

[B42] Easterbrooks M. A. Biringen Z. (2000). Guest editors’ introduction to the special issue: mapping the terrain of emotional availability and attachment. *Attach. Hum. Dev.* 2 123–129 10.1080/14616730050085518 11707906

[B43] Easterbrooks M. A. Biringen Z. (2005). The emotional availability scales: methodological refinements of the construct and clinical implications related to gender and at-risk interactions. *Infant Ment. Health J.* 26 291–294 10.1002/imhj.20053 28682467

[B44] Easterbrooks M. A. Chaudhuri J. H. Cestsdottir S. (2005). Patterns of emotional availability among young mothers and their infants: a dyadic, contextual analysis. *Infant Ment. Health J.* 26 309–326 10.1002/imhj.20057 28682449

[B45] Edhborg M. Lundh W. Seimyr L. Widstrom A. M. (2001). The long-term impact of postnatal depressed mood on mother–child interaction: a preliminary study. *J. Reprod. Infant Psychol.* 19 61–71 10.1080/02646830123255

[B46] Emde R. N. (1980). “Emotional availability: a reciprocal reward system for infants and parents with implications for prevention of psychosocial disorders,” in *Parent-Infant Relationships* ed. Taylor P. M. (Orlando, FL: Grune & Stratton)

[B47] Ensminger M. E. Fothergill K. E. (2003). “A decade of measuring SES: what it tells us and where to go from here,” in *Socioeconomic Status, Parenting, and Child Development* ed. Bornstein M. H. Bradley R. H. (Mahvah, NJ: Lawrence Erlbaum Associates) 13–27

[B48] Evans G. W. Gonnella C. Marcynyszyn L. A. Gentile L. Salpekar N. (2005). The role of chaos in poverty and children’s socioemotional adjustment. *Psychol. Sci.* 16 560–565 10.1111/j.0956-7976.2005.01575.x 16008790

[B49] Fergusson D. M. Boden J. M. Horwood L. J. (2007). Exposure to single parenthood in childhood and later mental health, educational, economic, and criminal behavior outcomes. *Arch. Gen. Psychiatry* 64 1089–1095 10.1001/archpsyc.64.9.1089 17768274

[B50] Fish M. (2001). Attachment in low-ses rural appalachian infants: contextual, infant, and maternal interaction risk and protective factors. *Infant Ment. Health J.* 22 641–664 10.1002/imhj.1024

[B51] Gazzotti S. Spinelli M. Albizzati A. Riva Crugnola C. (2010). Maternità in età adolescenziale: attaccamento materno, stili di interazione, stati affettivi, coordinazione affettiva madre-bambino. *Età Evol.* 96 64–74

[B52] Golombok S. Tasker F. Murray C. (1997). Children raised in fatherless families from infancy: family relationships and the socioemotional development of children of lesbian and single heterosexual mothers. *J. Child Psychol. Psychiatry* 38 783–792 10.1111/j.1469-7610.1997.tb01596.x 9363577

[B53] Goodman S. H. Gotlib I. H. (1999). Risk for psychopathology in the children of depressed mothers: a developmental model for understanding mechanisms of transmission. *Psychol. Rev.* 106 458–490 10.1037/0033-295X.106.3.458 10467895

[B54] Goodman S. H. Gotlib I. H. (eds). (2002). *Children of Depressed Parents: Mechanisms of Risk and Implications for Treatment*. Washington, DC: American Psychological Association

[B55] Gottfried A. W. Gottfried A. E. Bathurst K. Guerin D. W. Parramore M. M. (2003). *Socioeconomic Status, Parenting, and Child Development*. Mahwah, NJ: Laurence Erlbaum Associates 189–207

[B56] Greenberg M. T. Spelts M. L. DeKlyen M. (1993). The role of attachment in the early development of disruptive behavior problems. *Dev. Psychopathol.* 5 191–213 10.1017/S095457940000434X

[B57] Howes C. Hamilton C. E. (1992). Children’s relationships with caregivers: mothers and child care teachers. *Child Dev.* 63 859–866 10.2307/1131238 1505244

[B58] Hubbs-Tait L. Huges K. P. Culp A. M. Osofsky J. D. Hann D. M. Eberarhart-Wright A. (1996). Children of adolescent mothers: attachment representation, maternal depression, and later problems. *Am. J. Orthopsychiatry* 66 416–426 10.1037/h0080192 8827265

[B59] Keown L. J. Woodward L. J. Field J. (2001). Language development of pre-school children born to teenage mothers. *Infant Child Dev.* 10 129–145 10.1002/icd.282

[B60] Kogan N. Carter A. S. (1996). Mother-infant reengagement following the still-face: the role of maternal emotional availability in infant affect regulation. *Infant Beh. Dev.* 19 359–370 10.1016/S0163-6383(96)90034-X

[B61] Kohlhoff J. Barnett B. (2013). Parenting self-efficacy: links with maternal depression, infant behaviour and adult attachment. *Early. Hum. Dev.* 89 249–256 10.1016/j.earlhumdev.2013.01.008 23398731

[B62] Landry S. H. Smith K. E. Swank P. R. (2006). Responsive parenting: Establishing early foundations for social communication and problem solving. *Dev. Psychol.* 42 627–642 10.1037/0012-1649.42.4.627 16802896

[B63] Lemelin J. P. Tarabulsy G. M. Provost M. A. (2006). Preschool cognitive development from infant temperament, maternal sensitivity and psychosocial risk. *Merrill Palmer Q.* 52 779–806 10.1353/mpq.2006.0038

[B64] Letourneau N. L. Stewart M. J. Barnfather A. K. (2004). Adolescent mothers: support needs, resources, and support-education interventions. *J. Adolesc. Health* 35 509–525 10.1016/j.jadohealth.2004.01.007 15581532

[B65] Long M. S. (2009). Disorganized attachment relationships in infants of adolescent mothers and factors that may augment positive outcomes. *Adolescence* 44 621–633 19950873

[B66] Lyons-Ruth K. Alpern L. Repacholi B. (1993). Disorganized infant attachment classification and maternal psychosocial problems as predictors of hostile-aggressive behavior in the preschool classroom. *Child Dev.* 64 572–585 10.2307/1131270 8477635

[B67] Lyons-Ruth K. Connell D. Grunebaum H. Botein S. (1990). Infants at social risk: maternal depression and family support services as mediators of infant development and security of attachment. *Child Dev.* 61 85–98 10.2307/1131049 2307048 PMC7323915

[B68] Lyons-Ruth K. Repacholi B. McLeod S. Silva E. (1991). Disorganized attachment behavior in infancy: short-term stability, maternal and infant correlates, and risk-related subtypes. *Dev. Psychopathol.* 3 377–396 10.1017/S0954579400007586

[B69] Madigan S. Morgan G. Pederson D. R. (2006). Unresolved state of mind, disorganized attachment relationships, and disrupted interactions of adolescents mothers and their infants. *Dev. Psychol.* 42 293–304 10.1037/0012-1649.42.2.293 16569168

[B70] Markon K. E. Chmielewski M. Miller C. J. (2011). The reliability and validity of discrete and continuous measures of psychopathology: a quantitative review. *Psychol. Bull.* 137 856–879 10.1037/a0023678 21574681

[B71] Martins C. Gaffan E. A. (2000). Effects of early maternal depression on patterns of infant-mother attachment: a meta-analytic investigation. *J. Child Psychol. Psychiatry* 41 737–746 10.1111/1469-7610.00661 11039686

[B72] McCabe J. E. (2014). Maternal personality and psychopathology as determinants of parenting behavior: a quantitative integration of two parenting literatures. *Psychol. Bull.* 140 722–750 10.1037/a0034835 24295555

[B73] McGraw K. O. Wong S. P. (1996). Forming inferences about some intraclass correlation coefficients. *Psychol. Methods* 1 30–46 10.1037/1082-989X.1.1.30

[B74] McLoyd V. C. Wilson L. (1991). “The strain of living poor: parenting, social support, and child mental health,” in *Children in Poverty: Child Development and Public Policy* ed. Huston A. C. (Cambridge: Cambridge University Press) 105–135

[B75] Moran G. Forbes L. Evans E. Tarabulsy G. M. Madigan S. (2008). Both maternal sensitivity and atypical maternal behavior independently predict attachment security and disorganization in adolescent mother-infant relationships. *Infant Behav. Dev.* 31 321–325 10.1016/j.infbeh.2007.12.012 18282606

[B76] Murray L. (1992). The impact of postnatal depression on infant development. *J. Child Psychol. Psychiatry* 33 543–561 10.1111/j.1469-7610.1992.tb00890.x 1577898

[B77] Murray L. Sinclair D. Cooper P. Ducoumau P. Turner P. (1999). The socioemotional development of 5-year-old children of postnatally depressed mothers. *J. Child Psychol. Psychiatry* 40 1259–1271 10604404

[B78] Nagata M. Nagai Y. Sobajima H. Ando T. Honjo S. (2004). Depression in the early postpartum period and attachment to children – in mothers of NICU infants. *Infant Child Dev.* 13 93–110 10.1002/icd.339

[B79] NICHD Early Child Care Research Network. (1997). The effects of infant child care on infant–mother attachment security: results of the NICHD study of early child care. *Child Dev.* 68 860–879 10.1111/j.1467-8624.1997.tb01967.x 29106728

[B80] NICHD Early Child Care Research Network. (2006). Infant–mother attachment classification: risk and protection in relation to changing maternal caregiving quality. *Dev. Psychol.* 42 38–58 10.1037/0012-1649.42.1.38 16420117

[B81] Nievar M. A. Becker B. J. (2008). Sensitivity as a privileged predictor of attachment: a second perspective on De Wolff and van IJzendoorn’s meta-analysis. *Soc. Dev.* 17 102–114 10.1111/j.1467-9507.2007.00417.x

[B82] Pomerleau A. Scuccimarri C. Malcuit G. (2003). Mother-infant behavioral interactions in teenage and adult mothers during the first six months postpartum: relations with infant development. *Infant Men. Health J.* 24 495–509 10.1002/imhj.10073

[B83] Rafferty Y. Griffin K. W. Lodise M. (2011). Adolescent motherhood and developmental outcomes of children in early head start: the influence of maternal parenting behaviors, well-being, and risk factors within the family setting. *Am. J. Orthopsychiatry* 81 228–245 10.1111/j.1939-0025.2011.01092.x 21486265

[B84] Robinson J. L. Little C. Biringen Z. (1993). Emotional communication in mother-toddler relationships: evidence for early gender differentiation. *Merrill Palmer Q.* 39 496–517

[B85] Rosen D. Spencer M. S. Tolman R. M. Williams D. R. Jackson J. S. (2003). Psychiatric disorders and substance dependence among unmarried low-income mothers. *Health Soc. Work* 28 157–165 10.1093/hsw/28.2.157 12774537

[B86] Rosenkrantz Aronson S. Huston A. (2004). The mother-infant relationship in single, cohabiting, and married families: a case for marriage? *J. Fam. Psychol.* 18 5–18 10.1037/0893-3200.18.1.5 14992606

[B87] Sameroff A. J. (1998). Environmental risk factor in infancy. *Pediatrics* 102 1287–1292 9794971

[B88] Sameroff A. J. (2000). “Ecological perspectives on developmental risk,” in *WAIMH Handbook of Infant Mental Health: Vol. 5 Infant Mental Health in Groups at High Risk acura di* eds Osofsky J. D. Fitzgerald H. E. (New York: Wiley) 1–33

[B89] Scharte M. Bolte G. (2011). Impact of socioeconomic and environmental factors on increased health risks of children with single mothers. *Am. J. Epidemiol.* 173 S218–S218

[B90] Seifer R. Dickstein S. (2000). “Parental mental illness and infant development,” in *Handbook of Infant Mental Health* ed. Zeanah C. H. (New York: Guilford Press) 145–160

[B91] Seifer R. Schiller M. Sameroff A. J. Resnick S. Riordan K. (1996). Attachment, maternal sensitivity, and infant temperament during the first year of life. *Dev. Psychol.* 32 12–25 10.1037/0012-1649.32.1.12

[B92] Seow W. K. (2012). Environmental, maternal, and child factors which contribute to early childhood caries: a unifying conceptual model. *Int. J. Paediatr. Den.* 22 157–168 10.1111/j.1365-263X.2011.01186.x 21972925

[B93] Shaw D. Hyde L. V. Brennan L. L. (2012). Early predictors of boys’ antisocial trajectories. *Dev. Psychopathol.* 24 871–888 10.1017/S0954579412000429 22781860 PMC3409584

[B94] Shin H. Park Y. J. Kim M. J. (2006). Predictors of maternal sensitivity during the early postpartum period. *J. Adv. Nurs.* 55 425–434 10.1111/j.1365-2648.2006.03943.x 16866838

[B95] Sieger K. Renk K. (2007). Pregnant and parenting adolescents: a study of ethnic identity, emotional and behavioral functioning, child characeristics, and social support. *J. Youth Adolesc.* 36 567–581 10.1007/s10964-007-9182-6

[B96] Spieker S. J. Booth C. L. (1988). “Maternal antecedents of attachment quality,” in *Clinical Implications of Attachment* eds Belsky J. Nezworski T. (Hillsdale, NJ: Erlbaum) 95–135

[B97] Steele M. Steele H. Johansson M. (2002). Maternal predictors of children social cognition: an attachment perspective. *J. Child Psychol. Psychiatry* 43 861–872 10.1111/1469-7610.00096 12405475

[B98] Svoboda D. V. Shaw T. V. Barth R. P. Bright C. L. (2012). Pregnancy and parenting among youth in foster care: a review. *Child Youth Serv. Rev.* 34 867–875 10.1016/j.childyouth.2012.01.023

[B99] Tamis-LeMonda C. S. Bornstein M. H. Baumwell L. (2001). Maternal responsiveness and children’s achievement of language milestones. *Child Dev.* 72 748–767 10.1111/1467-8624.00313 11405580

[B100] Tarabulsy G. M. Bernier A. Provost M. A. Maranda J. Larose S. Moss E. (2005). Another look inside the gap: ecological contributions to the transmission of attachment in a sample of adolescent mother-infant dyads. *Dev. Psychol.* 41 212–224 10.1037/0012-1649.41.1.212 15656750

[B101] Teti D. M. Gelfand D. Messinger D. Isabella R. (1995). Maternal depression and the quality of early attachment: an examination of infants, preschoolers, and their mothers. *Dev. Psychol.* 31 364–376 10.1037/0012-1649.31.3.364

[B102] Teti D. M. Ablard K. E. (1989). Security of attachment and infant-sibling relationships: a laboratory study. *Child Dev.* 60 1519–1528 10.2307/1130940 2612257

[B103] Toth S. L. Rogosch F. A. Sturge-Apple M. Cicchetti D. (2009). Maternal depression, children’s attachment security, and representational development: an organizational perspective. *Child Dev.* 80 192–208 10.1111/j.1467-8624.2008.01254.x 19236401 PMC2785452

[B104] Tremblay R. E. Japel C. (2003). “Prevention during pregnancy, infancy and preschool years,” in *Early Prevention of Adult Antisocial Behaviour* eds Farrington D. P. Coid J. W. (Cambridge: Cambridge University Press)

[B105] van Bakel H. J. A. Riksen-Walraven J. M. (2002). Parenting and development of one-year-olds: links with parental, contextual and child characteristics. *Child Dev.* 73 256–273 10.1111/1467-8624.00404 14717256

[B106] van IJzendoorn M. H. Schuengel C. Bakersman-Kranenburg M. J. (1999). Disorganized attachment in early childhood: meta-analyses of precursors, concomitants, and sequelae. *Dev. Psychopathol.* 11 225–249 10.1017/S0954579499002035 16506532

[B107] Vaughn B. E. Waters E. (1990). Attachment behavior at home and in laboratory: q-sort observations and Strange Situation classi cation of one-year-olds. *Child Dev.* 61 1965–1973 10.2307/1130850 2083508

[B108] Vondra J. I. Shaw D. S. Swearingen L. Cohen M. Owens E. B. (2001). Attachment stability and emotional and behavioral regulation from infancy to preschool age. *Dev. Psychopathol.* 13 13–33 10.1017/S095457940100102X 11346048

[B109] Ward M. J. Carlson E. A. (1995). Associations among adult attachment representations, maternal sensitivity, and infant-mother attachment in a sample of adolescent mothers. *Child Dev.* 66 69–79 10.2307/1131191 7497830

[B110] Weinraub M. Horvath D. L. Gringlas M. B. (2002). “Single parenthood,” in *Handbook of Parenting: Being and Becoming a Parent* Vol. 3 ed. Bornstein M. H. (Mahwah, NJ: Lawrence Erlbaum Associates) 109–140

[B111] Weters E. (1987). *Attachment Behaviour Q-Set (Revision 3.0). Manoscritto non Pubblicato*. New York: State University of New York at Stony Brook

[B112] Yoshikawa H. Aber J. L. Beardslee W. R. (2012). The effects of poverty on the mental, emotional, and behavioral health of children and youth implications for prevention. *Am. Psychol.* 67 272–284 10.1037/a0028015 22583341

[B113] Zimmerman L. McDonald L. (1995). Emotional availability in infants’ relationships with multiple caregivers. *Am. J. Orthopsychiatry* 65 147–152 10.1037/h0079586 7733210

